# Association between Pre-Existing Sleep Disorders and Survival Rates of Patients with Breast Cancer

**DOI:** 10.3390/cancers14030798

**Published:** 2022-02-04

**Authors:** Yen-Chang Chen, Wan-Ming Chen, Ming-Feng Chiang, Ben-Chang Shia, Szu-Yuan Wu

**Affiliations:** 1 Division of Chest Medicine, Department of Internal Medicine, Lo-Hsu Medical Foundation, Lotung Poh-Ai Hospital, Yilan 265, Taiwan; dr.yenchang.chen@gmail.com; 2 Graduate Institute of Business Administration, College of Management, Fu Jen Catholic University, Taipei 242062, Taiwan; daisywanmingchen@gmail.com; 3 Division of Gastroenterology and Hepatology, Department of Internal Medicine, Lo-Hsu Medical Foundation, Lotung Poh-Ai Hospital, Yilan 265, Taiwan; chiangmingf@gmail.com; 4 Artificial Intelligence Development Center, Fu Jen Catholic University, Taipei 242062, Taiwan; 5 Department of Food Nutrition and Health Biotechnology, College of Medical and Health Science, Asia University, Taichung 413, Taiwan; 6 Big Data Center, Lo-Hsu Medical Foundation, Lotung Poh-Ai Hospital, Yilan 265, Taiwan; 7 Division of Radiation Oncology, Lo-Hsu Medical Foundation, Lotung Poh-Ai Hospital, Yilan 265, Taiwan; 8 Department of Healthcare Administration, College of Medical and Health Science, Asia University, Taichung 413, Taiwan; 9 Cancer Center, Lo-Hsu Medical Foundation, Lotung Poh-Ai Hospital, Yilan 265, Taiwan; 10 Centers for Regional Anesthesia and Pain Medicine, Taipei Municipal Wan Fang Hospital, Taipei Medical University, Taipei 110, Taiwan

**Keywords:** breast cancer, invasive ductal carcinoma, sleep disorder, survival, risk factor

## Abstract

**Simple Summary:**

This is the first study to estimate the effects of pre-existing sleep disorders on the survival outcomes of patients with breast cancer after receiving standard treatments. We conducted a head-to-head propensity score matching study to mimic a randomized trial to compare the survival rates of women with both sleep disorders and breast cancer. Women with pre-existing sleep disorders receiving curative treatments for breast cancer had poorer survival outcomes than those without sleep disorders. Therefore, patients should be screened and evaluated for pre-existing sleep disorders prior to breast surgery, with such disorders serving as survival predictors in patients with breast cancer. Future studies may investigate the survival benefits of pharmacological and behavioral treatments for sleep problems in patients with breast cancer.

**Abstract:**

PURPOSE: To investigate the effects of pre-existing sleep disorders on the survival outcomes of women receiving standard treatments for breast invasive ductal carcinoma (IDC). METHODS: We recruited patients from the Taiwan Cancer Registry Database who had received surgery for clinical stage I–III breast IDC. The Cox proportional hazards model was used to analyze all-cause mortality. We categorized the patients into those with and without sleep disorders (Groups 1 and 2, respectively) through propensity score matching. RESULTS: In the multivariate Cox regression analysis, the adjusted hazard ratio for all-cause mortality for Group 1 compared with Group 2 was 1.51 (95% confidence interval: 1.19, 1.91; *p* < 0.001). CONCLUSION: Our study demonstrated that the sleep disorder group had poorer survival rates than the non-sleep disorder group in breast cancer. Therefore, patients should be screened and evaluated for pre-existing sleep disorders prior to breast surgery, with such disorders serving as a predictor of survival in patients with breast cancer. Future studies may investigate the survival benefits of pharmacological and behavioral treatments for sleep problems in patients with breast cancer.

## 1. Introduction

Sleep disorders are prevalent in women with breast cancer. Approximately 60% to 90% of patients with breast cancer have sleep disturbances, which is a higher prevalence than that found in other types of cancer [1,2,3,4,5]. A recent meta-analysis that examined the sleep quality of women with breast cancer indicated that women with breast cancer already had sleep problems before receiving treatment [6]. Although their sleep disturbances might be ameliorated during the first months of treatment, they may be exacerbated with increased treatment time [6].

The association between sleep and the risk of all-cause mortality among the general population has received substantial attention from researchers [7,8,9,10]. Despite more than half of cancer survivors having sleep problems—a much higher prevalence than reported among the general population—knowledge regarding the association between sleep and cancer survival is relatively meager [11,12,13]. Although studies have determined that patients with breast cancer tend to have a higher mortality risk if they also have sleep problems prior to their breast cancer diagnosis, the data are controversial for sleep disorders and cancer survival [14,15]. These studies have reported inconsistent findings concerning the influence of sleep quality on the risk of death in patients with breast cancer [14,15]. These disparities indicate that additional studies should be conducted to examine the association between sleep and mortality risk for patients with breast cancer, with findings possibly helping to improve their quality of life and long-term survival rates.

Our study was conducted to assess whether a pre-existing sleep disorder is an independent risk factor for mortality in women with invasive ductal carcinoma (IDC) who underwent breast surgery followed by adjuvant treatments stipulated in the National Comprehensive Cancer Network (NCCN) guidelines [16]. By identifying the association between pre-existing sleep disorders and the survival of women with IDC, we expect to elucidate the matter of sleep disorders among women with IDC and provide an evidence-based basis for future research that may improve the survival of patients with sleep disorders; in particular, health-care providers stand to benefit from our findings. If patients who do not have a diagnosis of a sleep disorder before undergoing breast surgery exhibited a superior survival, routine screenings for sleep disorders and offering appropriate treatment for women who are at risk of having breast cancer may be beneficial. Interventions by health-care providers that facilitate the discovery of and lead to treatments for sleep disorders in these individuals will improve their chances of survival if they receive a diagnosis of IDC.

## 2. Patients and Methods

### 2.1. Study Population

The patient data analyzed in this study was sourced from the Taiwan Cancer Registry Database (TCRD). The TCRD is a nationwide registry detailing information on each patient’s cancer status and stage, cigarette smoking habit, treatment modalities, pathologic data, irradiation doses, and chemotherapy regimen used [17,18]. The database also keeps specific information for breast cancer, including hormone receptor and human epidermal growth factor receptor 2 (HER2) statuses [17,18]. Patients diagnosed with American Joint Committee on Cancer (AJCC) clinical stage I–III breast IDC between 1 January 2009 and 31 December 2018 were enrolled. The index date was the date of breast surgery. The duration of follow-up was measured from the index date to 31 December 2019. Our protocols were reviewed and approved by the Institutional Review Board of Taipei Medical University (TMU-JIRB No. 201712019).

### 2.2. Inclusion and Exclusion Criteria

The pathological information of all patients enrolled in this study was reviewed to ensure that all patients had newly diagnosed breast IDC and no other cancers or distant metastases. The women who were eligible for this study were at least 20 years old and had a diagnosis of IDC at a clinical stage of I–III, according to the *AJCC Cancer Staging Manual*, 7th Edition [19].

We excluded patients with a history of cancer before receiving a diagnosis of IDC, unclear staging, histological types other than IDC, or an unclear Charlson comorbidity index (CCI) and those without known pathological types or sex data [20,21]. Patients who had received nonstandard adjuvant breast irradiation (i.e., irradiation doses lower than 50 Gy to the chest wall, whole breast, or regional lymph nodes), an unclear histologic grade or tumor differentiation, missing hormone receptor status, missing HER2 status, or unclear staging were also excluded. Hormone receptor-positive tumors were defined as tumor cells in which at least 1% of the nuclei was stained positive for estrogen receptors and progesterone receptors through immunohistochemistry [22], and HER2-positive tumors were defined as those with immunohistochemistry staining of 3+ intensity or a HER2 gene-to-centromere 17 ratio, revealed by fluorescence in situ hybridization testing, of ≥2.0 [23]. Based on the NCCN guidelines adopted in Taiwan, adjuvant treatments, such as adjuvant radiotherapy (RT), chemotherapy, hormone therapy, or target therapy, did not disqualify patients from the study [16]. Patients who had unclear surgical procedures or ill-defined nodal surgery were excluded from our cohort.

The CCI was implemented to measure the incidence of comorbidities. The codes of the *International Classification of Diseases, 9th Revision, Clinical Modification* (ICD-9-CM) were applied to code comorbidities [24]. The CCI score only counted comorbidities which were observed within 12 months before the index date, and included comorbidities reported and assigned at the first admission or after more than two outpatient department visits. To prevent covariates from being adjusted repetitively in multivariate analysis, we also excluded diabetes, hyperlipidemia, end-stage renal disease (ESRD), liver cirrhosis, acute myocardial infarction (AMI), coronary artery disease (CAD), and stroke from the CCI scores.

Enrolled patients were categorized as having sleep disorders 1 year prior to the index date if an ICD (including ICD-9-CM) code corresponding to the third edition of the *International Classification of Sleep Disorders* (ICSD-3) [25] had been assigned in two or more family medicine, neurology, or psychology outpatient visits or at least one course of hospitalization. Patients were also deemed to have a sleep disorder if they had an ICD code corresponding to the ICSD-3 and had been prescribed benzodiazepines for at least a total of 30 days the year prior to the index date. The ICSD-3, which includes 60 specific diagnoses within seven categories, including insomnia disorders, sleep-related breathing disorders, central disorders of hypersomnolence, circadian rhythm sleep-wake disorders, sleep-related movement disorders, parasomnias, and other sleep disorders, is the primary reference used to diagnose and classify sleep disorders [26].

Our study included patients who received partial (breast-conserving surgery or breast conservative surgery [BCS]; i.e., lumpectomy) or total mastectomy for their IDC. Patients who underwent BCS were typically treated with moderate-dose RT after surgery to eradicate microscopic residual disease.

### 2.3. Propensity Score Matching and Covariates

Propensity score matching (PSM) was implemented to reduce confounding effects before comparing all-cause death between Groups 1 and 2. With a caliper width of 0.2, the two groups were matched at a ratio of 1:1 for the variables of age, income level, urbanization, menopausal status, HER2 status, AJCC clinical stage, CCI score, type of breast surgery, and the presence of diabetes, hyperlipidemia, ESRD, liver cirrhosis, AMI, CAD, stroke, hormone receptors, differentiation, nodal surgery, chemotherapy, and adjuvant RT [27]. A Cox regression model was used to regress all-cause mortality on various sleep disorder statuses, and a robust sandwich variance estimator was applied to account for clustering within matched sets [28]. Using the method of PSM, potential predictors were controlled prior to statistical analysis (Table 1). Through multivariate time-dependent Cox regression analysis, possible independent predictors of all-cause death were determined by calculating the hazard ratios (HRs) associated with sleep disorder status, age, income level, urbanization, menopausal status, HER2 status, AJCC clinical stage, CCI score, type of breast surgery, and the presence of diabetes, hyperlipidemia, ESRD, liver cirrhosis, AMI, CAD, stroke, hormone receptors, differentiation, nodal surgery, chemotherapy, and adjuvant RT. The primary endpoint for Groups 1 and 2 was all-cause mortality.

After application of the inclusion and exclusion criteria and use of PSM, we identified 2966 patients with breast IDC who had undergone breast surgery and an SLNB or ALND for AJCC clinical stage I–III IDC. These patients were eligible for further analysis and, in order to compare all-cause mortality, were divided into two groups based on their sleep disorder status. Patients with a sleep disorder diagnosed before breast surgery were placed into Group 1, and those without a diagnosis of a pre-existing sleep disorder were placed into Group 2.

### 2.4. Statistics

Independent t-tests and a chi-square test were implemented to compare continuous variables and categorical variables, respectively, between Groups 1 and 2. Continuous variables were summarized and described as mean ± standard deviation. Differences in follow-up time between the two groups were compared with the Mann–Whitney U test. The *p* values for adjuvant RT and chemotherapy were generated using Gray’s test (Table 1). A *p* value is considered significant if it is less than 0.05 in a two-tailed Wald test. Comparative analyses were performed after confounders were adjusted. Estimated with the Kaplan–Meier method, differences in survival curves between the two groups were stratified according to matched sets and compared using a stratified logrank test [29]. SAS version 9.4 (SAS Institute, Cary, NC, USA) was used for statistical analyses in this study.

## 3. Results

### 3.1. Propensity Score Matching and Study Cohort

A total of 2966 patients (1483 patients in each group matched by PSM) with stage I–IIIB-C IDC were eligible for further analysis based on the inclusion and exclusion criteria. The clinicodemographic characteristics of each group are summarized in Table 1. Because of PSM, there were no statistically significant differences in these characteristics, namely age, income level, urbanization, menopausal status, HER2 status, AJCC clinical stage, CCI score, type of breast surgery, and the presence of diabetes, hyperlipidemia, ESRD, liver cirrhosis, AMI, CAD, stroke, hormone receptors, differentiation of tumor, nodal surgery, chemotherapy, and adjuvant RT. Differences between the two groups were noted in death, follow-up duration, and sleep disorder status within 1 year before the index date.

### 3.2. Prognostic Factors of All-Cause Death

The multivariate Cox regression for survival analyses showed poorer overall survival (OS) in patients with a sleep disorder, old age (>65 years), a high CCI score, hormone receptor negative status, premenopause, a high grade of differentiation, and an advanced AJCC clinical stage (Table 2). Comorbidities such as diabetes, hyperlipidemia, ESRD, liver cirrhosis, AMI, CAD, stroke, income levels, urbanization, methods of nodal or breast surgery, chemotherapy, or adjuvant chemotherapy did not show significant differences (Table 2). Calculated through multivariate survival analysis using Cox regression model, the adjusted HR (aHR) of all-cause death for patients with sleep disorders compared with those without sleep disorders was 1.51 (95% confidence interval [CI]: 1.19, 1.91; *p* < 0.001). The aHRs of all-cause death were 1.81 (95% CI: 1.39, 2.25) for those aged between 65 years and 75 years, 6.11 (95% CI: 4.40, 8.48) for those aged between 76 years and 85 years, and 13.97 (95% CI: 8.05, 24.24) for those aged older than 85 years, compared with those aged 65 years or younger. The aHR was 2.02 (95% CI: 1.53, 2.66) for those with a CCI of ≥1, compared with those with a CCI of zero. The aHR for patients with a negative hormone receptor was 0.86 (95% CI: 0.50, 0.97), compared with those with a positive hormone receptor. The aHR was 1.13 (95% CI: 1.06, 1.22) for patients in premenopause, compared with those in postmenopause. The aHRs of patients with a differentiation of Grade II was 1.17 (95% CI: 1.05, 1.53) and 1.33 (95% CI: 1.04, 2.44) for those with a differentiation of Grade III, compared with those with a differentiation of Grade I. The aHRs were 1.13 (95% CI: 1.01, 2.17), 1.19 (95% CI: 1.04, 1.87), and 1.92 (95% CI: 1.56, 2.53) for patients with AJCC clinical stages II, IIIA, and IIIB–C, respectively, compared with those with an AJCC clinical stage of I.

### 3.3. Kaplan–Meier OS for Patients with IDC with and without Sleep Disorders before Breast Surgery

**Figure 1:**
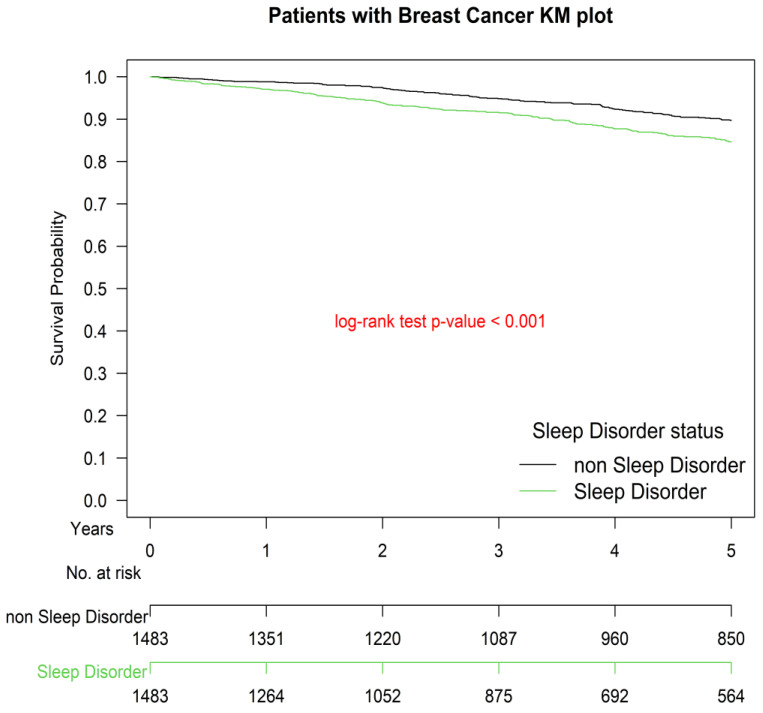
Kaplan–Meier survival curves of propensity score-matched patients with breast cancer with and without sleep disorders.

The Kaplan–Meier survival curve for patients with IDC and a sleep disorder diagnosed before breast surgery was inferior to that of those without a sleep disorder (*p* < 0.001; Figure 1).

## 4. Discussion

This study revealed a significant relationship between pre-existing sleep disorders and breast cancer mortality. Compared with patients without a sleep disorder, those who had been diagnosed with a sleep disorder within the year before breast cancer surgery had an increased risk of all-cause death. To the best of our knowledge, no previous head-to-head PSM study estimated the OS rates for patients with breast cancer with pre-existing sleep disorders after they received surgery and standard treatments according to the *Clinical Practice Guidelines in Oncology* (NCCN Guidelines) [30]. Our study is the first head-to-head PSM that imitates a randomized trial to balance all the confounding factors listed in Table 1.

As indicated in our study, a sleep disorder diagnosed 1 year prior to breast surgery is an independent risk factor for the OS of patients diagnosed with stage I–III breast cancer and receiving standard treatments (Table 2; Figure 1). Based on the literature reviewed in this study, rather than focusing on pre-existing or pretreatment sleep disorders, almost all studies were conducted to assess the risk of all-cause death posed by sleep problems that were reported by the patients after receiving a diagnosis of breast cancer. However, given that most sleep disorders begin before a breast cancer diagnosis and precancerous sleep problems tend to linger and become aggravated during cancer treatment [6,12,31], our findings may still be deemed consistent with the findings of studies that reported sleep problems as a risk factor for breast cancer mortality [14,15,32,33]. In addition to these major findings, our study also corroborates the findings of studies that have identified age, comorbidity, hormone receptor status, menopausal status, grade of differentiation, AJCC pathologic stages, and HER2 status as independent predictors of OS (Table 2) [34,35,36,37,38]. Although the significance of the results presented in Table 2 should be considered in future clinical practice and trials concerning breast cancer and sleep disorders, further studies may be required to establish the mechanism behind our findings.

Several factors may influence the cause or mechanism of the association between sleep disorders and the increase in all-cause death in women with breast IDC receiving standard treatments. First, a study determined that women hospitalized with breast cancer experienced more complications and were hospitalized longer if they also had a diagnosis of a sleep disorder [39]. Second, studies have indicated that sleep inconsistency and duration are associated with an increased risk of recurrence and progression in survivors of breast cancer and an increased risk of developing more aggressive breast cancer, respectively [32,33,40]. According to the consensus of the American Academy of Sleep Medicine, a sleep duration of less than 7 h is associated with abnormal immune function and an increased risk of death [41]. Third, sleep disturbance and long sleep duration are associated with increased systemic biomarkers of inflammation, such as C-reactive protein (CRP), interleukin 6 (IL-6), and fibrinogen [42,43]; systemic inflammation is a vital contributor to tumorigenesis, and medication that regulates inflammation, such as statins, may suppress tumor growth and increase the chance of survival [44,45]. Compared with their male counterparts, women with subjective sleep problems may have an increased elevation of inflammatory biomarkers [46,47]. The association between systemic inflammation and breast cancer survival has also been widely studied over the past two decades. Recent studies have suggested that increased pretreatment with CRP concentrations has been linked to a higher recurrence and poorer survival in women with breast cancer [48,49,50,51]. Elevated pretreatment plasma fibrinogen levels were associated with reduced rates of pathological complete response to neoadjuvant chemotherapy, 3- and 5-year disease-free survival rates, and OS rates in patients with breast cancer [52,53], and high serum levels of IL-6 were associated with tumor metastasis and patient survival [51,54].

A sleep disorder diagnosis 1 year before breast surgery is an independent prognostic factor for the survival of patients with IDC after breast surgery. This is a major finding because most patients with breast cancer and sleep problems received their diagnosis of a sleep disorder before receiving a diagnosis of breast cancer [6,12,31]. Because patients without a diagnosis of a sleep disorder before undergoing breast surgery exhibited a superior OS rate (Table 2; Figure 1), routine screenings for sleep disorders and offering appropriate treatment for women who are at risk of having breast cancer may be beneficial. Interventions that facilitate the discovery of and lead to treatments for sleep disorders in these individuals will improve their chances of survival if they receive a diagnosis of IDC. Additionally, an improvement in sleep for women diagnosed with sleep disorders and those at high risk of the development of breast cancer can potentially improve their overall survival if they go on to develop breast cancer in the future.

Our study has several strengths. It is the first and largest cohort study to estimate the effects of pre-existing sleep disorders on the survival outcomes of patients with IDC after undergoing standard breast treatments in accordance with NCCN guidelines. Additionally, from an oncology perspective, this study highlights the necessity of sleep quality, for which a prospective, randomized controlled study is unlikely to be conducted without ethical concerns. We also analyzed most prognostic factors identified in the literature for OS in patients with IDC if the data were available in the TCRD. Because PSM minimized the effect of confounding, the covariates between the groups with and without sleep disorders were homogenous for patients with stage I–III breast cancer receiving breast surgery and standard treatments, and no selection bias was noted between the two groups (Table 1).

We recognize some limitations of our study. First, as in all retrospective studies, residual or unmeasured confounding and selection bias are inevitable. Second, the TCRD does not include information, such as health behavior, level of education, family support, or body mass index, that might also have an impact on survival. Third, enrolled patients with sleep disorders were not further stratified by the seven categories identified by the ICSD-3. The degree of influence of each sleep disorder subtype on the OS of IDC patients were not analyzed respectively in our study. Fourth, ethnic differences in sleep complaints, prevalence of sleep disorder, and survival of patient with breast cancer have been reported in previous studies [55,56,57,58]. Therefore, extrapolation of our results to non-Asian populations must be done with caution given that the patients enrolled in this study were from an Asian population. Finally, the significance of the results of our study is subject to the quality and accuracy of the data recorded in the TCRD. Nevertheless, because the Taiwan Cancer Registry Administration periodically audits files and punishes hospitals for which there is evidence of abnormal charges or improper practices identified through medical chart reviews and patient interviews, the TCR long-form data have most likely maintained accuracy to a high standard [59]. Therefore, our conclusions are unlikely to be affected by these limitations, considering that the results presented a great magnitude of statistical significance.

## 5. Conclusions

Our study revealed that the OS of IDC patients who had undergone breast surgery was worse in those with pre-existing sleep disorders than those without sleep disorders. Therefore, we suggest screening all patients diagnosed with IDC for pre-existing sleep disorders as this information may impact overall survival of patients undergoing breast surgery. Treatment of pre-existing sleep disorders may provide survival benefits in such patients.

## Figures and Tables

**Table 1:** Characteristics of propensity score-matched patients with and without sleep disorders before a diagnosis of breast cancer.

Variables	Without Sleep Disorder		With Sleep Disorder		*p* Value
	N = 1483	(100%)	N = 1483	(100%)	
Age (mean ± SD)	(56.51 ± 11.79)		(56.56 ± 11.85)		0.415
Age groups					0.137
Age ≤ 65 years	1183	79.77%	1134	76.47%	
65 years < age ≤ 75 years	14	0.94%	21	1.42%	
75 years < age ≤ 85 years	208	14.03%	233	15.71%	
Age > 85 years	78	5.26%	95	6.41%	
CCI Score					0.055
=0	1333	89.89%	1299	87.59%	
≥1	150	10.11%	184	12.41%	
Diabetes					0.742
No	1208	81.46%	1200	80.92%	
Yes	275	18.54%	283	19.08%	
Hyperlipidemia					0.121
No	1118	75.39%	1080	72.83%	
Yes	365	24.61%	403	27.17%	
ESRD					0.501
No	1475	99.46%	1471	99.19%	
Yes	8	0.54%	12	0.81%	
Liver cirrhosis					0.188
No	1163	78.42%	1132	76.33%	
Yes	320	21.58%	351	23.67%	
AMI					0.741
No	1466	98.85%	1463	98.65%	
Yes	17	1.15%	20	1.35%	
CAD					0.138
No	1413	95.28%	1386	93.46%	
Yes	70	4.72%	97	6.54%	
Stroke					0.114
No	1438	96.97%	1430	96.43%	
Yes	45	3.03%	53	3.57%	
Income level (NTD/month)					0.465
Low income	51	3.44%	59	3.98%	
Income ≤ 20,000	677	45.65%	703	47.40%	
20,000 < income ≤ 30,000	383	25.83%	350	23.60%	
Income > 30,000	372	25.08%	371	25.02%	
Urbanization					0.616
Rural	309	20.84%	297	20.03%	
Urban	1174	79.16%	1186	79.97%	
Hormone receptor					0.992
Negative	370	24.95%	373	25.15%	
Positive	1113	75.05%	1110	74.85%	
Menopausal status					0.931
No	888	59.88%	893	60.22%	
Yes	595	40.12%	590	39.78%	
Differentiation					0.754
I	222	14.97%	224	15.10%	
II	669	45.11%	671	45.25%	
III	592	39.92%	588	39.65%	
AJCC clinical stage					0.893
I	691	46.59%	690	46.53%	
II	345	23.26%	355	23.94%	
IIIA	358	24.14%	350	23.61%	
IIIB-C	89	6.00%	88	5.93%	
HER2 status					0.914
Negative	1186	79.97%	1193	80.45%	
Positive	297	20.03%	290	19.55%	
Nodal surgery					0.962
ALND	443	29.87%	440	29.67%	
SLB	1040	70.13%	1043	70.33%	
Breast surgery					0.951
BCS	1255	84.63%	1250	84.29%	
Total mastectomy	228	15.37%	233	15.71%	
Chemotherapy					0.525
No	607	40.93%	589	39.72%	
Yes	876	59.07%	894	60.28%	
Adjuvant radiotherapy					0.979
No	208	14.03%	211	14.23%	
Yes	1275	85.97%	1272	85.77%	
Follow-up, years, Median (IQR; Q1, Q3)	5.88 (2.74, 9.90)		3.74 (1.64, 6.67)		<0.001
Follow-up, years (mean ± SD)	(6.68 ± 4.42)		(4.52 ± 3.50)		<0.001
Death					<0.001
No	1264	85.23%	1214	81.86%	
Yes	219	14.77%	269	18.14%	

AJCC, American Joint Committee on Cancer; HER2, human epidermal growth factor receptor 2; ESRD, end-stage renal disease; AMI, acute myocardial infarction; CAD, coronary artery disease; BCS, breast conservative surgery; SLNB, sentinel lymph node biopsy; ALND, axillary lymph node dissection; NTD, New Taiwan Dollars; SD, standard deviation; IQR, interquartile range; CCI, Charlson comorbidity index.

**Table 2:** Univariate and multivariate Cox proportional regression of all-cause mortality between patients with breast cancer with and without sleep disorders.

Variables	Crude HR (95% CI)		Adjusted HR (95% CI) *		*p* Value
Sleep disorder status (ref.: Nonsleep disorder)					
Sleep disorder	1.42	(1.18, 1.71)	1.51	(1.19, 1.91)	<0.001
Age (ref.: Age ≤ 65 years)					
65 years < age ≤ 75 years	1.88	(1.49, 2.38)	1.81	(1.39, 2.35)	<0.001
75 years < age ≤ 85 years	4.61	(3.51, 6.06)	6.11	(4.40, 8.48)	<0.001
Age > 85 years	9.65	(5.89, 15.8)	13.97	(8.05, 24.24)	<0.001
CCI score (ref.: 0)					
≥1	2.39	(1.90, 3.01)	2.02	(1.53, 2.66)	<0.001
Diabetes (ref.: No)					
Yes	1.07	(0.60, 1.43)	1.13	(0.72, 1.95)	0.761
Hyperlipidemia (ref.: No)					
Yes	1.18	(0.95, 1.47)	0.72	(0.56, 1.92)	0.609
ESRD (ref.: No)					
Yes	1.75	(0.78, 2.92)	1.10	(0.46, 1.61)	0.826
Liver cirrhosis (ref.: No)					
Yes	1.24	(1.0, 1.55)	1.14	(0.74, 1.29)	0.607
AMI (ref.: No)					
Yes	1.42	(0.96, 2.31)	1.22	(0.90, 2.30)	0.250
CAD (ref.: Not)					
Yes	1.24	(0.84, 1.52)	1.17	(0.84, 1.43)	0.264
Stroke (ref.: Not)					
Yes	1.28	(0.52, 2.41)	1.01	(0.59, 1.74)	0.969
Income (ref.: >$NT30,000)					
20,000 < income ≤ 30,000	1.17	(0.74, 1.33)	1.05	(0.84, 2.01)	0.328
Income ≤ 20,000	1.32	(0.76, 2.07)	1.22	(0.55, 1.44)	0.664
Low income	2.23	(0.80, 3.21)	2.05	(0.88, 3.03)	0.462
Urbanization (ref.: Rural)					
Urban	0.86	(0.69, 1.07)	0.95	(0.75, 1.19)	0.637
Hormone receptor (ref.: Negative)					
Positive	0.57	(0.21, 0.84)	0.86	(0.50, 0.97)	0.007
Menopausal status (ref.: Postmenopausal)					
Premenopausal	1.16	(1.17, 1.84)	1.13	(1.06, 1.22)	0.037
Differentiation (ref.: Differentiation grade I)					
II	1.61	(1.16, 2.23)	1.17	(1.05, 1.53)	0.027
III	1.29	(0.93, 2.07)	1.33	(1.04, 2.44)	0.019
AJCC clinical stage (ref.: Stage I)					
II	1.14	(1.09, 2.52)	1.13	(1.01, 2.17)	0.044
IIIA	1.23	(1.08, 2.70)	1.19	(1.04, 1.87)	0.027
IIIB–C	1.69	(1.04, 2.73)	1.92	(1.56, 2.53)	0.002
HER2 status (ref.: Negative)					
Positive	1.08	(1.02, 1.55)	1.05	(1.01, 1.29)	0.014
Nodal surgery (ref.: ALND)					
SLB	1.14	(0.79, 1.63)	0.69	(0.43, 1.11)	0.127
Breast surgery (ref.: BCS)					0.951
Total mastectomy	1.14	(0.87, 1.50)	0.90	(0.65, 1.24)	0.507
Chemotherapy (ref.: No)					
Yes	1.55	(0.98, 2.45)	1.43	(0.78, 2.62)	0.250
Adjuvant radiotherapy (ref.: No)					
Yes	1.35	(0.90, 2.05)	1.16	(0.61, 2.19)	0.658

HR, hazard ratio; CI, confidence interval; AJCC, American Joint Committee on Cancer; HER2, human epidermal growth factor receptor 2; ESRD, end-stage renal disease; AMI, acute myocardial infarction; CAD, coronary artery disease; BCS, breast conservative surgery; SLNB, sentinel lymph node biopsy; ALND, axillary lymph node dissection; NTD, New Taiwan Dollars; ref., reference group; CCI, Charlson comorbidity index. * All covariates mentioned in Table 2 were adjusted.

## Data Availability

The data sets supporting the study conclusions are included in this manuscript.

## Abbreviations

aHR: adjusted hazard ratio; CI, confidence interval; AJCC, American Joint Committee on Cancer; TCRD, Taiwan Cancer Registry Database; PSM, propensity score matching; OS, overall survival; CRP, C-reactive protein; IL, interleukin; CCI, Charlson comorbidity index; NCCN, National Comprehensive Cancer Network; IDC, invasive ductal carcinoma; HER2, human epidermal growth factor receptor 2; *ICD-9-CM*, *International Classification of Diseases, 9th Revision*, *Clinical Modification*; ESRD, end-stage renal disease; AMI, acute myocardial infarction; CAD, coronary artery disease; ICSD-3, *International Classification of Sleep Disorders*; BCS, breast conservative surgery; SLNB, sentinel lymph node biopsy; ALND, axillary lymph node dissection.

## References

[1] Otte J.L., Davis L., Carpenter J.S., Krier C., Skaar T.C., Rand K.L., Weaver M., Landis C., Chernyak Y., Manchanda S. (2016). Sleep disorders in breast cancer survivors. Support. Care Cancer.

[2] Williams S.A., Schreier A.M. (2005). The role of education in managing fatigue, anxiety, and sleep disorders in women undergoing chemotherapy for breast cancer. Appl. Nurs. Res..

[3] Jacob L., Scholten P.C., Kostev K., Kalder M. (2018). Association between sleep disorders and the presence of breast cancer metastases in gynecological practices in Germany: A case—Control study of 11,412 women. Breast Cancer Res. Treat..

[4] Eldin E.-S.T., Younis S.G., El Aziz L.M.A., Eldin A.T., Erfan S.T. (2019). Evaluation of sleep pattern disorders in breast cancer patients receiving adjuvant treatment (chemotherapy and/or radiotherapy) using polysomnography. J. BUON Off. J. Balk. Union Oncol..

[5] Savard J., Ivers H., Villa J., Caplette-Gingras A., Morin C.M. (2011). Natural Course of Insomnia Comorbid with Cancer: An 18-Month Longitudinal Study. J. Clin. Oncol..

[6] Chang W.-P., Chang Y.-P. (2019). Meta-Analysis of Changes in Sleep Quality of Women with Breast Cancer before and after Therapy. Breast Care.

[7] Dew M.A., Hoch C.C., Buysse D.J., Monk T.H., Begley A.E., Houck P.R., Hall M., Kupfer D.J., Reynolds C.F. (2003). Healthy Older Adults’ Sleep Predicts All-Cause Mortality at 4 to 19 Years of Follow-Up. Psychosom. Med..

[8] Ferrie J.E., Shipley M.J., Cappuccio F.P., Brunner E., A Miller M., Kumari M., Marmot M. (2007). A Prospective Study of Change in Sleep Duration: Associations with Mortality in the Whitehall II Cohort. Sleep.

[9] Cappuccio F.P., D’Elia L., Strazzullo P., Miller M.A. (2010). Sleep Duration and All-Cause Mortality: A Systematic Review and Meta-Analysis of Prospective Studies. Sleep.

[10] Shen X., Wu Y., Zhang D. (2016). Nighttime sleep duration, 24-hour sleep duration and risk of all-cause mortality among adults: A meta-analysis of prospective cohort studies. Sci. Rep..

[11] Savard J., Morin C.M. (2001). Insomnia in the Context of Cancer: A Review of a Neglected Problem. J. Clin. Oncol..

[12] Savard J., Simard S., Blanchet J., Ivers H., Morin C.M. (2001). Prevalence, Clinical Characteristics, and Risk Factors for Insomnia in the Context of Breast Cancer. Sleep.

[13] Mystakidou K., Parpa E., Tsilika E., Pathiaki M., Patiraki E., Galanos A., Vlahos L. (2007). Sleep quality in advanced cancer patients. J. Psychosom. Res..

[14] Phipps A.I., Bhatti P., Neuhouser M.L., Chen C., Crane T.E., Kroenke C.H., Ochs-Balcom H., Rissling M., Snively B.M., Stefanick M.L. (2016). Pre-diagnostic Sleep Duration and Sleep Quality in Relation to Subsequent Cancer Survival. J. Clin. Sleep Med..

[15] Trudel-Fitzgerald C., Zhou E., Poole E.M., Zhang X., Michels K.B., Eliassen A.H., Chen W.Y., Holmes M.D., Tworoger S.S., Schernhammer E. (2017). Sleep and survival among women with breast cancer: 30 years of follow-up within the Nurses’ Health Study. Br. J. Cancer.

[16] Gradishar W.J., Moran M.S., Abraham J., Aft R., Agnese D., Allison K.H., Blair S.L., Burstein H.J., Dang C., Elias A.D. (2021). NCCN Guidelines® Insights: Breast Cancer, Version 4.2021. J. Natl. Compr. Cancer Netw..

[17] Zhang J., Lu C.-Y., Chen H.-M., Wu S.-Y. (2021). Neoadjuvant Chemotherapy or Endocrine Therapy for Invasive Ductal Carcinoma of the Breast with High Hormone Receptor Positivity and Human Epidermal Growth Factor Receptor 2 Negativity. JAMA Netw. Open.

[18] Zhang J., Lu C.-Y., Qin L., Chen H.-M., Wu S.-Y. (2020). Breast-conserving surgery with or without irradiation in women with invasive ductal carcinoma of the breast receiving preoperative systemic therapy: A cohort study. Breast.

[19] Hari D.M., Leung A.M., Lee J.-H., Sim M.-S., Vuong B., Chiu C.G., Bilchik A.J. (2013). AJCC Cancer Staging Manual 7th edition criteria for colon cancer: Do the complex modifications improve prognostic assessment?. J. Am. Coll. Surg..

[20] Charlson M., Szatrowski T.P., Peterson J., Gold J. (1994). Validation of a combined comorbidity index. J. Clin. Epidemiol..

[21] Sundararajan V., Henderson T., Perry C., Muggivan A., Quan H., Ghali W.A. (2004). New ICD-10 version of the Charlson comorbidity index predicted in-hospital mortality. J. Clin. Epidemiol..

[22] Hammond M.E.H., Hayes D.F., Dowsett M., Allred D.C., Hagerty K.L., Badve S., Fitzgibbons P.L., Francis G., Goldstein N.S., Hayes M. (2010). American Society of Clinical Oncology/College of American Pathologists Guideline Recommendations for Immunohistochemical Testing of Estrogen and Progesterone Receptors in Breast Cancer. J. Clin. Oncol..

[23] Fehrenbacher L., Cecchini R.S., Jr C.E.G., Rastogi P., Costantino J.P., Atkins J.N., Crown J.P., Polikoff J., Boileau J.-F., Provencher L. (2020). NSABP B-47/NRG Oncology Phase III Randomized Trial Comparing Adjuvant Chemotherapy with or Without Trastuzumab in High-Risk Invasive Breast Cancer Negative for HER2 by FISH and With IHC 1+ or 2+. J. Clin. Oncol..

[24] Geraci J.M., Ashton C.M., Kuykendall D.H., Johnson M.L., Wu L. (1997). International Classification of Diseases, 9th Revision, Clinical Modification Codes in Discharge Abstracts Are Poor Measures of Complication Occurrence in Medical Inpatients. Med Care.

[25] Sateia M.J. (2014). International Classification of Sleep Disorders.

[26] Sateia M.J. (2014). International Classification of Sleep Disorders-Third Edition. Chest.

[27] Austin P.C. (2011). Optimal caliper widths for propensity-score matching when estimating differences in means and differences in proportions in observational studies. Pharm. Stat..

[28] Austin P.C. (2012). The performance of different propensity score methods for estimating marginal hazard ratios. Stat. Med..

[29] Austin P.C. (2013). The use of propensity score methods with survival or time-to-event outcomes: Reporting measures of effect similar to those used in randomized experiments. Stat. Med..

[30] National Comprehensive Cancer Network NCCN Guidelines Version 3.2019 Breast Cancer. https://www.nccn.org/professionals/physician_gls/pdf/breast.pdf.

[31] Harris B., Ross J., Sanchez-Reilly S. (2014). Sleeping in the Arms of Cancer. Cancer J..

[32] Mansano-Schlosser T.C., Ceolim M.F. (2017). Association between poor clinical prognosis and sleep duration among breast cancer patients 1. Rev. Lat.-Am. De Enferm..

[33] Marinac C.R., Nelson S.H., Flatt S.W., Natarajan L., Pierce J.P., Patterson R.E. (2017). Sleep duration and breast cancer prognosis: Perspectives from the Women’s Healthy Eating and Living Study. Breast Cancer Res. Treat..

[34] Rakha E.A., Reis-Filho J.S., Baehner F., Dabbs D.J., Decker T., Eusebi V., Fox S.B., Ichihara S., Jacquemier J., Lakhani S.R. (2010). Breast cancer prognostic classification in the molecular era: The role of histological grade. Breast Cancer Res..

[35] Dong G., Wang D., Liang X., Gao H., Wang L., Yu X., Liu J. (2014). Factors related to survival rates for breast cancer patients. Int. J. Clin. Exp. Med..

[36] Hong T.V., Ba D.N., Skoog L., Thanh V.T., Tani E. (2019). Breast Cancer Survival Defined by Biological Receptor and Menopausal Status in Vietnamese Women. Cancer Control.

[37] Parise C.A., Caggiano V. (2020). The influence of comorbidity on treatment and survival of triple-negative breast cancer. Breast J..

[38] Pan H., Wang H., Qian M., Mao X., Shi G., Ma G., Yu M., Xie H., Ling L., Ding Q. (2021). Comparison of Survival Outcomes Among Patients with Breast Cancer with Distant vs Ipsilateral Supraclavicular Lymph Node Metastases. JAMA Netw. Open.

[39] Vin-Raviv N., Akinyemiju T.F., Galea S., Bovbjerg D.H. (2017). Sleep disorder diagnoses and clinical outcomes among hospitalized breast cancer patients: A nationwide inpatient sample study. Support. Care Cancer.

[40] Thompson C.L., Li L. (2012). Association of sleep duration and breast cancer OncotypeDX recurrence score. Breast Cancer Res. Treat..

[41] Watson N.F., Badr M.S., Belenky G., Bliwise D.L., Buxton O., Buysse D., Dinges D.F., Gangwisch J., Grandner M.A., Kushida C. (2015). Recommended Amount of Sleep for a Healthy Adult: A Joint Consensus Statement of the American Academy of Sleep Medicine and Sleep Research Society. Sleep.

[42] Dowd J.B., Goldman N., Weinstein M. (2011). Sleep Duration, Sleep Quality, and Biomarkers of Inflammation in a Taiwanese Population. Ann. Epidemiol..

[43] Irwin M.R., Olmstead R., Carroll J. (2015). Sleep Disturbance, Sleep Duration, and Inflammation: A Systematic Review and Meta-Analysis of Cohort Studies and Experimental Sleep Deprivation. Biol. Psychiatry.

[44] Peek R.M., Mohla S., Dubois R.N. (2005). Inflammation in the Genesis and Perpetuation of Cancer: Summary and Recommendations from a National Cancer Institute—Sponsored Meeting. Cancer Res..

[45] Van Gestel Y.R.B.M., E Hoeks S., Sin D.D., Huzeir V., Stam H., Mertens F.W., van Domburg R.T., Bax J.J., Poldermans D. (2009). COPD and cancer mortality: The influence of statins. Thorax.

[46] Suarez E.C. (2008). Self-reported symptoms of sleep disturbance and inflammation, coagulation, insulin resistance and psychosocial distress: Evidence for gender disparity. Brain Behav. Immun..

[47] Miller M.A., Kandala N.-B., Kivimaki M., Kumari M., Brunner E.J., Lowe G.D., Marmot M.G., Cappuccio F.P. (2009). Gender Differences in the Cross-Sectional Relationships Between Sleep Duration and Markers of inflammation: Whitehall II Study. Sleep.

[48] Asegaonkar S.B., Asegaonkar B.N., Takalkar U.V., Advani S., Thorat A.P. (2015). C-Reactive Protein and Breast Cancer: New Insights from Old Molecule. Int. J. Breast Cancer.

[49] Allin K.H., Nordestgaard B.G., Flyger H., E Bojesen S. (2011). Elevated pre-treatment levels of plasma C-reactive protein are associated with poor prognosis after breast cancer: A cohort study. Breast Cancer Res..

[50] Liu X., Guo X., Zhang Z. (2021). Preoperative Serum Hypersensitive-c-Reactive-Protein (Hs-CRP) to Albumin Ratio Predicts Survival in Patients with Luminal B Subtype Breast Cancer. OncoTargets Ther..

[51] Shimura T., Shibata M., Gonda K., Murakami Y., Noda M., Tachibana K., Abe N., Ohtake T. (2019). Prognostic impact of interleukin-6 and C-reactive protein on patients with breast cancer. Oncol. Lett..

[52] Mei Y., Zhao S., Lu X., Liu H., Li X., Ma R. (2016). Clinical and Prognostic Significance of Preoperative Plasma Fibrinogen Levels in Patients with Operable Breast Cancer. PLoS ONE.

[53] Wang Y., Wang Y., Chen R., Tang Z., Peng Y., Jin Y., Lan A., Ding N., Dai Y., Jiang L. (2021). Plasma fibrinogen acts as a predictive factor for pathological complete response to neoadjuvant chemotherapy in breast cancer: A retrospective study of 1004 Chinese breast cancer patients. BMC Cancer.

[54] Dethlefsen C., Højfeldt G., Hojman P. (2013). The role of intratumoral and systemic IL-6 in breast cancer. Breast Cancer Res. Treat..

[55] Johnson D.A., Jackson C.L., Williams N.J., Alcántara C. (2019). Are sleep patterns influenced by race/ethnicity—A marker of relative advantage or disadvantage? Evidence to date. Nat. Sci. Sleep.

[56] Lim D.W., Giannakeas V., Narod S.A. (2020). Survival Differences in Chinese Versus White Women with Breast Cancer in the United States: A SEER-Based Analysis. JCO Glob. Oncol..

[57] Chen X., Wang R., Zee P., Lutsey P.L., Javaheri S., Alcántara C., Jackson C., Williams M.A., Redline S. (2015). Racial/Ethnic Differences in Sleep Disturbances: The Multi-Ethnic Study of Atherosclerosis (MESA). Sleep.

[58] Tin S.T., Elwood J.M., Brown C., Sarfati D., Campbell I., Scott N., Ramsaroop R., Seneviratne S., Harvey V., Lawrenson R. (2018). Ethnic disparities in breast cancer survival in New Zealand: Which factors contribute?. BMC Cancer.

[59] Kao C.W., Chiang C.J., Lin L.J., Huang C.W., Lee W.C., Lee M.Y., the Taiwan Society of Cancer Registry Expert Group (2021). Accuracy of long-form data in the Taiwan cancer registry. J. Formos. Med. Assoc..

